# Experimental renal transplantation in rats improves cardiac dysfunction caused by chronic kidney disease while LVH persists

**DOI:** 10.3389/fcvm.2023.1200323

**Published:** 2023-06-29

**Authors:** Linda Hagmayer, Christina Mayer, Nadja Ebert, Kerstin Amann, Christoph Daniel

**Affiliations:** Department of Nephropathology, Friedrich-Alexander University (FAU) Erlangen-Nuremberg, Erlangen, Germany

**Keywords:** chronic kidney disease, cardiac hypertrophy, FGF23, fibrosis, renal tranplantation

## Abstract

**Background:**

Chronic kidney disease (CKD) causes congestive heart failure (CHF) with systolic dysfunction and left ventricular hypertrophy (LVH), which is a major contributor to increased mortality in CKD patients. It remains unclear whether cardiovascular changes that occur during the course of CKD can be reversed when renal function is restored by transplantation.

**Methods:**

To investigate this, chronic kidney disease was established in F344 rats by subtotal nephrectomy (SNx) for 8 weeks, followed by transplantation of a functional kidney from an isogenic F344 donor. SNx rats without transplantation and sham-operated animals served as controls. Renal function was assessed before and throughout the experiment. In addition, cardiac ultrasound was performed at weeks 0, 8, 12 and 16. At the end of the experiment, intra-arterial blood pressure was measured and kidneys and hearts were histologically and molecularly examined.

**Results:**

Eight weeks after SNx, rats developed marked renal dysfunction associated with significant glomerulosclerosis and tubulointerstitial fibrosis, but also an increase in left ventricular mass. After transplantation, renal function normalized but relative heart weight and ventricular mass as assessed by ultrasound scans showed no reduction compared with SNx controls. However, left ventricular wall thickness, fractional shortening and ejection fraction was normalized by renal transplantation. At 8 weeks after kidney transplantation, cardiac expression of BNP and FGF23 was also at levels comparable to healthy controls, whereas these factors were significantly increased in SNx rats. Cardiac fibrosis, as measured by fibronectin mRNA expression, was completely normalized, whereas cardiac fibronectin protein was still slightly but not significantly increased in transplanted animals compared to controls. In addition, the myofibroblast marker collagen 1, as assessed by immunohistochemistry, was significantly increased in SNx rats and also normalized by renal transplantation. Interestingly, CD68+ macrophages were significantly reduced in the hearts of SNx rats and in transplanted animals at slightly higher levels compared to controls.

**Conclusion:**

Restoration of renal function by kidney transplantation normalized early cardiac changes at most functional and molecular levels, but did not completely reverse LVH. However, further studies are needed to determine whether restoration of renal function can also reverse LVH at a later time point.

## 1. Introduction

In patients with chronic kidney disease (CKD), the disproportionately high prevalence and mortality of cardiovascular disease (CVD) is a major clinical problem (1).CVD is approximately 20 times more common in these patients than in age- and sex-matched normal populations and up to 3 times more common than in other high-risk populations, such as patients with diabetes mellitus (2). This is particularly true in young patients with CKD, who have up to a 1,000-fold increased cardiovascular risk (3). Numerous studies in non-dialysed CKD patients have demonstrated the importance of CKD as an independent cardiovascular risk factor even after adjustment for classic risk factors such as hypertension, diabetes and dyslipidemia (4). Contrary to earlier assumptions that the cause of cardiac death was exclusively due to coronary events and accelerated atherosclerosis, recent studies show that a significant proportion of cardiac mortality (up to 60%) is due to sudden cardiac death (5). Characteristic myocardial structural changes such as left ventricular hypertrophy (LVH), interstitial myocardial fibrosis and wall thickening of intramyocardial arteries represent a possible cause. The latter is associated with increased intercapillary distances and decreased blood and oxygen supply, contributing to the apparent decreased ischemic tolerance of the myocardium in CKD (6–8). The pathomechanisms responsible for CKD-specific myocardial structural changes are not fully understood. An important mediator in the pathogenesis of LVH appears to be fibroblast growth factor 23 (FGF-23), which can be induced by both high phosphate and inflammatory stimuli (9, 10). Improvement of renal function, after renal transplantation (RTx), should result in the absence of the processes and stimuli that trigger CKD-mediated LVH. There are few studies, some of them conflicting, on the extent and changes of cardiovascular changes after kidney transplantation. Despite several reports of improved LVH after successful RTx (11–14), others could not confirm this observation (15, 16). Therefore, it is unclear whether RTx can halt the disease progression or even reverse existing structural or functional changes and what molecular processes control this. In particular, it is not possible to study expression of hypertrophy-inducing genes, fibrosis and inflammation in the heart in RTx patients. Because of the close analogy of both CKD and CVD to the human situation and the possibility to perform RTx in the rat model, the subtotally nephrectomized (SNx) rat model is well suited to study this question (17).

## 2. Material and methods

### 2.1. Experimental design

**Figure 1:**
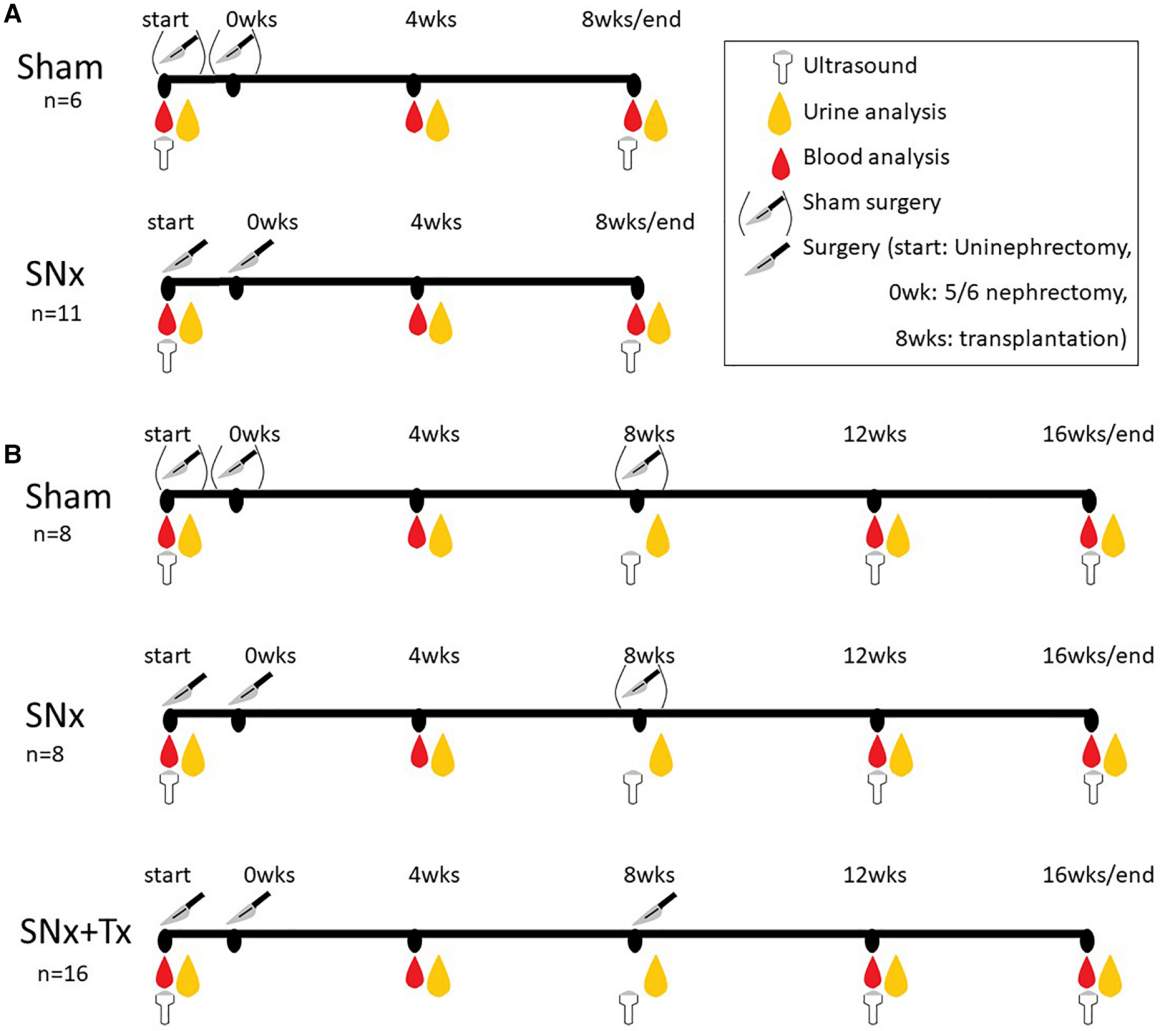
Experimental design of the study. SNx, 5/6 nephrectomy; Tx, kidney transplantation.

All animal experiments were performed in accordance with ARRIVE guidelines (18) and EU Directive 2010/63/EU on the care and use of laboratory animals. The experimental protocol for the animal experiments was approved by the German Regional Committee for the Care and Use of Animals, which is equivalent to the US IACUC, and approved by the Government of Middle Franconia (approval number: 54-2532.1-52/12) before the animal experiments were performed in strict compliance with the German Animal Welfare Act. Male F344 rats (Charles River, Sulzfeld, Germany) weighing 200–250 g at 8 weeks of age were used in all animal experiments and fed standard rat chow (Rat & Mouse Standard Diet, Sniff Spezialdiäten GmbH, Soest, Germany) and tap water *ad libitum*. A control group of 6 animals (sham) and a 5/6 nephrectomy (SNx) group of 11 animals were used to study renal and cardiac changes after 8 weeks of SNx (Figure 1A). For the 16-week experiment to study the reversibility of cardiovascular changes we used another sham control group (group 1; *n* = 8), while 28 animals with SNx were randomized into two groups according to their levels of proteinuria. In group 2 the rats with SNx received no further treatment (*n* = 12, of which 4 died before the end of the study due to the severity of their kidney disease and could not be included in the study). The group 3 comprised SNx at the start of the study followed by RTx at week 8 (*n* = 16).

#### 2.1.1. Subtotal nephrectomy (SNx)

For surgery, the rats were anesthetized by inhalation anesthesia with isoflurane, the incision site was shaved and disinfected, and the animal was placed in lateral position. The abdomen was opened through a lateral incision at the level of the kidney, which was previously anesthetized with a topical application of xylocaine spray (Aspen Germany GmbH). During the first surgical session, the right kidney was carefully decapsulated for preservation of the adrenal gland before the kidney was completely removed. Then, in the second surgery 1 week later, SNx of the left kidney is performed. For SNx, the upper and lower poles of the remaining kidney are resected as previously described (19). Bleeding is controlled by compression. To determine the amount of resected tissue, the kidney removed in the first surgery was weighed. The renal tissue resected in the second operation was then defined as 2/3 of the uni-nephrectomized kidney. Overall, this results in a resection of 5/6 of the original kidney tissue present in the rats (19). Wound closure was performed with a single layer of absorbable suture. The animals were analgesically treated with Buprenovet (Richter Pharma AG) at a dose of 0.3 mg/kg BID. The rats in the sham group were also anesthetized and the abdomen was opened without further surgical intervention to match the conditions of all groups.

#### 2.1.2. Renal transplantation (RTx)

For RTx the left kidney was exposed in the Fisher F344 donor rats, the renal vein was cut proximal to the vena cava, and the kidney was washed and preserved in ice-cold Custodiol® solution (Dr. Franz Köhler Chemie GmbH, Bensheim, Germany). The renal artery was used to create an aortic patch, and the ureter was excised next to the bladder. The kidneys were transplanted heterotopically with end-to-side anastomosis to the recipient's aorta and vena cava, respectively. The ureter was introduced into the bladder. Cold ischemia lasted approximately 50 min; warm ischemia lasted an average of 35 min. In the RTx experimental group, the native SNx kidney was removed from 3 rats during transplantation. Because the SNx kidney were often fused with other organs such as the spleen, leading to complications during removal, the native SNx kidney was left in place in the 9 RTx group rats. There were no significant differences in the parameters studied between the two surgical procedures, so both groups were combined into one RTx group.

### 2.2. Evaluation of renal function

Serum samples for measuring urea and 24 h urine collections for quantification of proteinuria were obtained in periodic intervals. Serum creatinine and urea were assessed using an auto analyzer (Beckman Instruments, Brea, CA, USA). After collection of 24 h urine using metabolic cages (Techniplast GmbH, Hohenpeißenberg, Germany) proteinuria was measured in urine samples diluted 1:10 in phosphate buffered saline (PBS) pH 7.6 (Sigma Aldrich Chemie GmbH, Steinheim, Germany) using the BioRad Protein Assay (BioRad, München, Germany). BSA (Pierce, Bonn, Germany) was used as standard in concentrations ranging from 0 to 1,500 µg/ml following manufactures instructions.

### 2.3. Evaluation of kidney injury

Renal biopsies were fixed in 4% paraformaldehyde (PFA) buffered in PBS pH 7.4, embedded in paraffin, and cut into sections of 1 µm for periodic acid Schiff reagent (PAS) staining. For each biopsy, 40 cortical glomerular cross-sections and 20 high power fields were evaluated in a blinded fashion by two independent observers at 400× magnification. Glomerulosclerosis and tubulointerstitial injury was graded semi-quantitatively using the glomerulosclerosis index (GSI) and tubulointerstitial injury (TI) score as previously described (20).

### 2.4. Evaluation of heart weight, morphology and function using high resolution ultrasound

To perform ultrasound-examination animals were anaesthetized using isoflurane. The rat was placed supine on a heating plate and the body temperature was maintained at a constant 37°C using a heatable operating table equipped with a rectal probe. The heart was examined using a Vevo 2,100 high-resolution ultrasound system equipped with an MS-250 microscan transducer and Vevolab software (both FUJIFILM Visualsonics, Toronto, Canada). To quantify the cardiac dimensions, the hearts were displayed in the short and long axes. A B-mode and M-mode display was selected for both slice planes. To perform the evaluations, the image data were analyzed on the computer using Vevolab software. During the analysis, the dimensions, especially wall thickness and ventricular diameter, are determined, as well as functional parameters that reflect the pumping capacity. M-mode measurements were performed in the parasternal long axis and short axis using 3–5 complexes each. A linear measurement comparing systole and diastole during a complex was performed as well as a myocardial measurement showing the total myocardial volume and ventricular volume during cardiac activity. All measurements were performed during both systole and diastole in apnea. To determine the exact time of measurement, systole and diastole were determined by ECG and a respiratory curve was created.

At the end of the experiment, the hearts were taken from the sacrificed animals and weighed. The left ventricle was then weighed after removal of the right ventricle and atrium. The relative heart weight and relative ventricle weight were determined by dividing each by the body weight of rats.

### 2.5. Histologic evaluation of heart and immunohistochemistry

For the histological examinations the tissue was fixed in 4% paraformaldehyde (PFA) in phosphate buffered saline (PBS) pH 7.4 or zinc-fixative (0.1 M Tris-buffer pH 7.4 supplemented with 3.2 mM calcium acetate, 37.8 mM zinc chloride, 27.3 mM zinc acetate). PFA fixed heart tissue was stained with periodic acid-Schiff reaction (PAS) or hematoxylin eosin (HE) for morphological analysis using standard routine protocol. Wall thicknesses of the left ventricles were measured on HE-stained sections at 4 measurement points each using Zen software (Zeiss, Oberkochen, Germany) and then averaged. In addition, the occurrence of microscarring in the perivascular space was analyzed in collagen 1 stained heart sections and graded semi-quantitatively: score 0 = nor scarring, 1 = weak scarring, 2 = moderate scarring, 3 = severe scarring. For detection of fibronectin and macrophages using immunohistochemistry, the slides were deparaffinized and rehydrated. Antigen retrieval was performed by cooking for 2.5–5 min in TRS (Target retrieval solution, DAKO Deutschland GmbH, Hamburg, Germany) using a decloaking chamber (Biocare Medical, Pacheco, CA, USA) followed by washing in 50 mM Tris(hydroxymethyl)aminomethan pH 7.6 supplemented with 0.1% Tween 20 (Tris-buffer). For collagen 1 staining, zinc-fixed heart section were used without antigen retrieval. Endogenous peroxidases was blocked with 3% H_2_O_2_ and unspecific binding by 20% normal horse serum (NHS, Vector Laboratories, Burlingame, CA, USA) and 5% milk powder diluted in Tris-buffer. The primary antibodies were applied in Tris-buffer containing 1% bovine serum albumin (BSA, Merck KGaA, Darmstadt, Germany) and were incubated overnight at 4°C. The following primary antibodies were used: a polyclonal rabbit anti-fibronectin antibody (ab2413, Abcam, Cambridge, GB); a polyclonal rabbit anti-collagen 1 antibody (Novus Biologicals, LLC, Centennial, CO, USA) and a monoclonal mouse anti-ED1 antibody (Bio-Rad Laboratories GmbH, Feldkirchen, Germany). After another wash with Tris-buffer, the secondary antibodies (a biotinylated horse anti-mouse IgG and a biotinylated goat anti-rabbit IgG; both from Vector Laboratories) were applied in Tris-buffer containing 1% BSA and incubated for 30 min at room temperature. Detection of bound antibodies was performed using the ABC kit and DAB-Immpact as substrate (both Vector Laboratories). After washing, slides were counterstained with haemalaun and covered by entellan (Merck KGaA). For the evaluation of immunohistochemical staining in cardiac sections, 10 visual fields were analyzed at 400× magnification. Finally, for ED1 staining, the mean number of ED1-positive macrophages per visual field was calculated and presented. Fibronectin (FN) and collagen 1 quantification in the heart was performed using a semiquantitative score: score 0 = no staining; score 1 = up to 25% of the visual field is positive; score 2 = up to 26%–50% positive; score 3 = up to 51%–75% positive; and score 4 = more than 75% of the visual field is positive. The mean FN and collagen 1 score was determined for each case.

### 2.6. FGF23-ELISA

An enzyme-linked immunosorbent assay (ELISA) for the detection of mouse/rat-FGF-23-(Intact) (Immunotopics Inc., San Clemente, CA, USA) was used to quantify FGF-23 in serum samples. Serum samples were aliquoted and diluted in steps of 1:10 using the sample diluent supplied with the ELISA-KIT. The ELISA was carried out following the manufacturer's instructions and using 20 μl of the diluted serum samples. Finally, substrate turnover was measured at a wavelength of 405 nm and a reference wavelength of 650 nm using a Synergy 2 microplate reader and Gen5.1 software (both BioTek Instruments GmbH, Bad Friedrichshall, Germany) and FGF-23 concentration was determined using a standard series.

### 2.7. Quantitative real-time PCR

To evaluate changes in relative mRNA expression levels at the endpoint of week 16, cardiac samples were collected from sham, SNx rats, and SNx rats that received a kidney transplant at week 8. Total mRNA was isolated using RNeasy Fibrous Tissue Mini columns (Qiagen, Hilden, Germany). Primers for the target genes were designed using the primer design software Primer Express 3 (Applied Biosystems, Weiterstadt, Germany) and synthesized (MWG-BIOTECH AG, Ebersberg, Germany) or collected from the literature (Table 1). Primers were tested for target specificity and amplification efficiency according to standard quality protocols provided by Applied Biosystems (Weiterstadt, Germany). Reverse transcription reactions and real-time PCR were performed using Power SYBR Green on a 7,500 Fast Real-time PCR System (both Applied Biosystems, Weiterstadt, Germany) according to the manufacturer's instructions. Real-time PCR data were analyzed using SDS v1.3 software (Applied Biosystems). To compare expression levels between groups, the relative expression of target gene mRNA levels was calculated using the comparative delta Ct (threshold cycle number) method (4). Normalization was performed against endogenous 18 S rRNA levels by applying the resulting relative fold changes.

**Table 1:** Primers used for real-time PCR.

No.	Target	fw	Rev
1	18 s	TTGATTAAGTCCCTGCCCTTTGT	CGATCCGAGGGCCTCACTA
2	fibronectin	TTGCAACCCACCGTGGAGTATGTG	CTCGGTAGCCAGTGAGCTTAACAC
3	CTGF	TGTGCACTGCCAAAGATGGT	GGTACACGGACCCACCGA
4	FGF23	ATCTCCGCGGCAACATTTT	AGGTAGACGTCGTAGCCGTTCT
5	FGFR4	CATTAGCCCATACAGCTCTG	CAGTCAAGTGGATGGCTC
6	BNP	GCTGTGACGGGCTGAGGT	GCCGCAGGCAGAGTCAGA
7	IL-6	TGAAACCCTAGTTCATATCTTCAAACA	AGCCACTCCTTCTGTGACTCTAACTT

### 2.8. Statistical analysis

After testing for normality using the Kolmogorov–Smirnov test, statistical significances (*p* < 0.05) were evaluated using Kruskal–Wallis Test followed by Dunn's multiple comparison test (GraphPad Prism software version 8) or Mann–Whitney *U* rank-test for comparison of two groups. Results are shown as box plots, showing the 25–75 percentile within the box and minimum and maximum values as whiskers.

## 3. Results

### 3.1. SNx induced chronic kidney disease in rats

**Figure 2:**
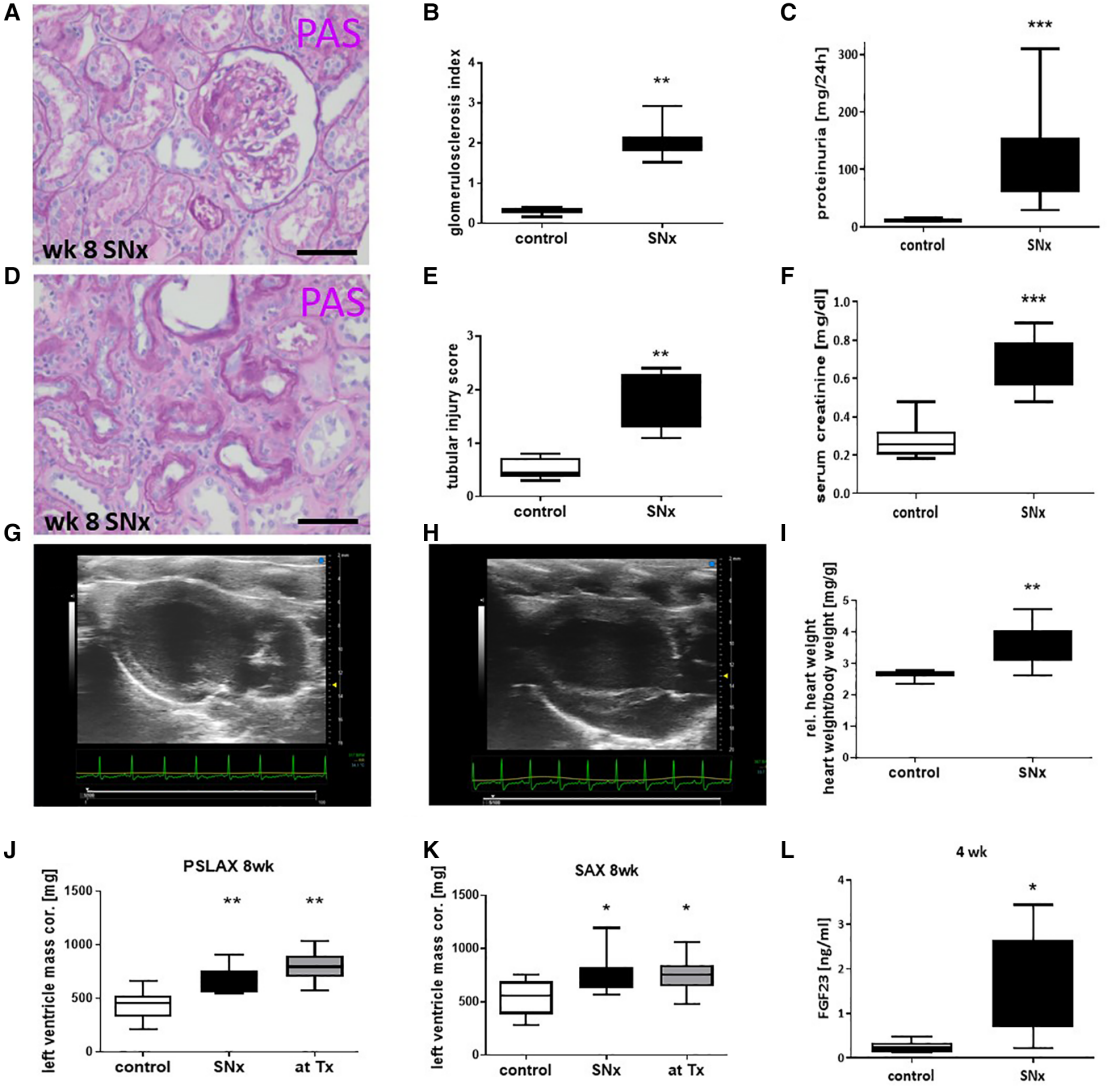
Snx induced a kidney disease and cardiac hypertrophy. Representative paraffin-embedded perjodate acid Schiff's (PAS) stained renal sections from glomeruli (**A**) and tubulointerstitial compartment (**D**) of SNx rats on week 8 were shown and evaluated for glomerulosclerosis (**B**) and tubular injury (**E**). Kidney function was measured by proteinuria (**C**) and serum creatinine (**F**). Examples of ultrasonic pictures from a heart of a sham (**G**) and a SNx rat (**H**) on week 8 are shown. Relative heart weight was compared in control and SNx rats 8 weeks after model induction (**I**). Left ventricle mass was evaluated using long axis (PSLAX, **J**) and short axis (**K**, SAX) view. Serum samples were evaluated for FGF23 levels using ELISA (**L**). Bar in A and D represents 50 µm. *p < 0.05 vs. control, **p < 0.01 vs. control, ***p < 0.001 vs. control.

Eight weeks after SNx, the reduction in renal mass resulted in significant morphological changes in the remaining renal tissue. The glomeruli showed significant sclerosis in the PAS staining (GSI; Figures 2A,B), which was accompanied by an approximately 8-fold increase in proteinuria (Figure 2C). In addition, significant tubulointerstitial damage with tubular atrophy, fibrosis, and an interstitial inflammatory response was observed in the kidneys at this time point (Figure 2D), which was analyzed using a tubulointerstitial injury score (TSI) (Figure 2E). Serum creatinine, a surrogate marker of renal function, was also significantly elevated (Figure 2F).

### 3.2. SNx induced cardiac hypertrophy and impaired the cardiac function

**Figure 3:**
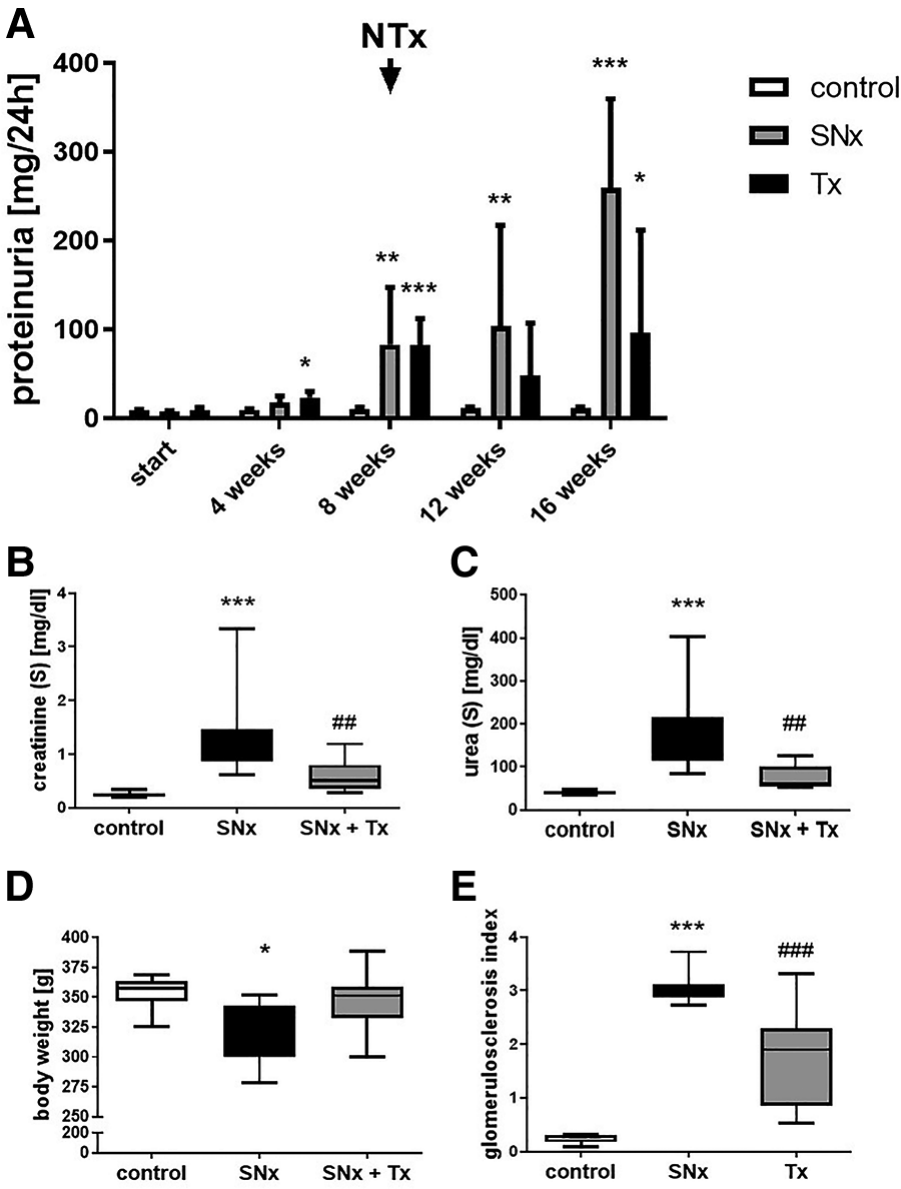
Kidney transplantation improves kidney function in SNx rats. Proteinuria was detected in (i) sham, (ii) SNx and (iii) SNx rats followed by renal transplantation at different time points during the experiment (**A**). At the end of experiment (week 16) serum creatinine (**B**), serum urea (**C**), body weight (**D**) and glomerulosclerosis (**E**) were analyzed in all groups. *p < 0.05 vs. control, **p < 0.01 vs. control, ***p < 0.001 vs. control, ^##^*p* < 0.01 vs. SNx, ^###^*p* < 0.001 vs. SNx.

Eight weeks after induction of SNx, we observed significant changes not only in the kidneys but also in the hearts of the rats. Echocardiography revealed a thickening of the left ventricle in SNx animals (Figure 2G) compared to sham controls (Figure 2H). Cardiac hypertrophy was also reflected in a significant increase in absolute (not shown) and relative heart weight (Figure 2I) and in the evaluation of echocardiographic measurements of left ventricular mass (Figures 2J, K). In addition, we observed a significant 6-fold increase of the hypertrophy-inducing factor FGF23 in the blood of the animals (Figure 2L). While proteinuria in the SNx group continued to increase throughout the study until the 16-week endpoint, proteinuria in the RTx group was lower at week 12 (4 weeks after RTx) than at the time of RTx and increased only slightly until the endpoint (Figure 3A). In SNx animals RTx was able to significantly normalize renal function, measured as serum creatinine and serum urea, compared to the SNx group, whereas it was slightly but not significantly increased compared to the sham group (Figures 3B,C). Also the body weight of the rats normalized after kidney transplantation (Figure 3D). Apparently, low-grade glomerulosclerosis was present in the grafts after RTx which may be a reason that the renal function failed to be fully restored (Figure 3E).

### 3.3. Renal transplantation reversed most SNx-induced cardiac changes but did not completely normalize LVH

**Figure 4:**
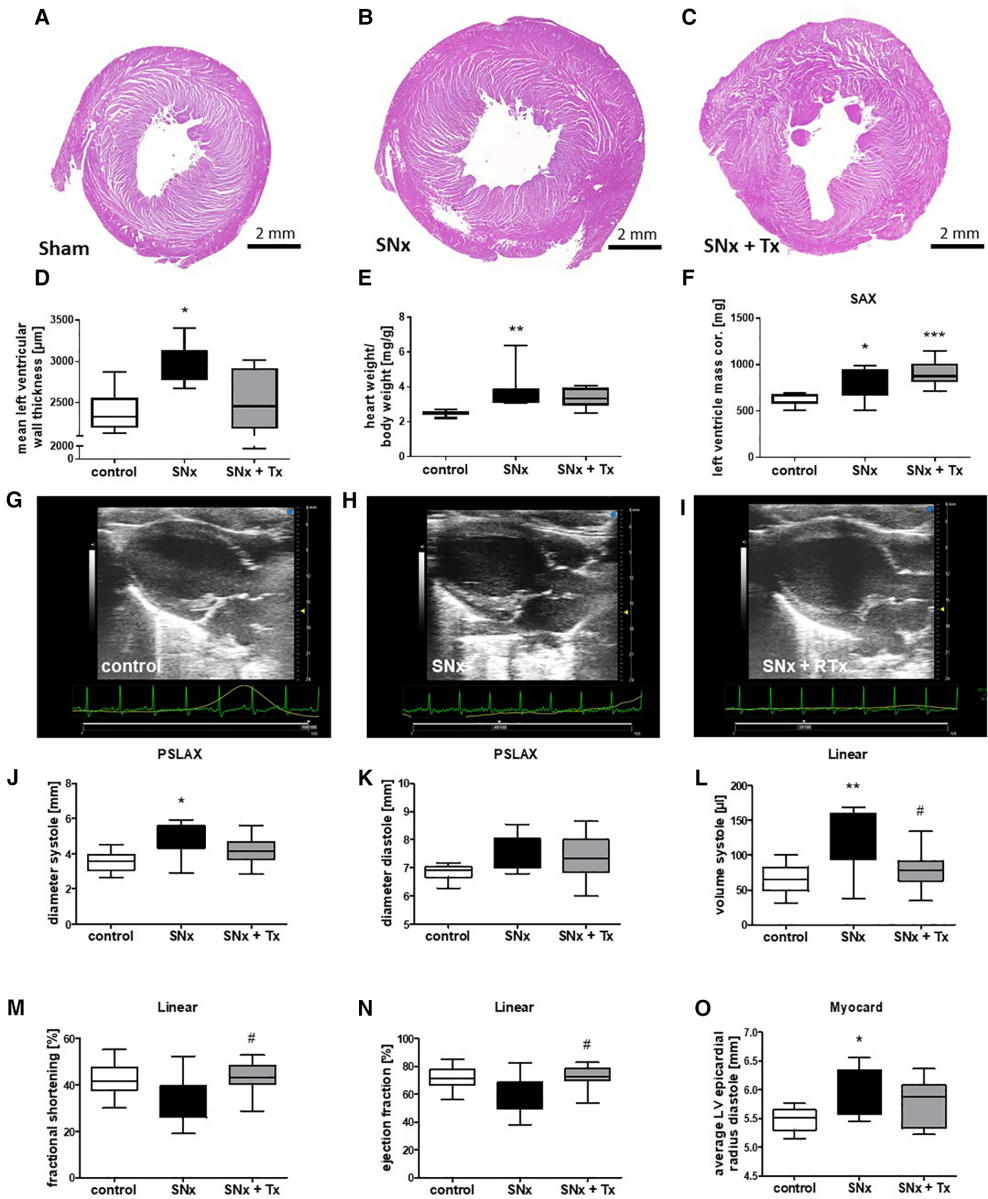
CKD induced cardiac dysfunction is reversible while LVH was unchanged 8 weeks after kidney transplantation. Representative images of HE-stained sections taken from hearts at the endpoint (week 16) are shown for sham (**A**), SNx (**B**), and SNx followed by renal transplantation (RTx) groups (**C**). LVH was assessed by measurement of left ventricular wall thickness using hematoxylin-stained paraffin sections (**D**), calculation of relative heart weight (**E**) and sonographical evaluation of left ventricular mass monitored on short axis (**F**). Representative echo-cardiographic images taken at the endpoint (week 16) are shown for the sham (**G**), SNx (**H**), and SNx followed by renal transplantation (RTx) groups (**I**). In the longitudinal axis echocardiography was used for measurement of systolic (**J**) and diastolic diameter (**K**). In the linear analysis systolic volume (**L**), fractional shortening (**M**) and ejection fraction were analyzed (**N**). Average LV epicardial radius diastole was measured in the heart (**O**). *p < 0.05 vs. control, **p < 0.01 vs. control, ***p < 0.001 vs. control, ^#^*p* < 0.05 vs. SNx.

LVH after SNx was not significantly improved by RTx in all analyses used to determine LVH. Analysis of left ventricular wall thickness measured on HE-stained heart sections at the end point (week 16) showed that the left ventricular wall was significantly thickened in the SNx group compared with the sham group (Figures 4A,B,D), but was reduced in SNx rats that received kidney transplantation (Figures 4C,D). In contrast, determination of relative heart weight (Figure 4E) and echocardiographic assessment of ventricular mass showed that LVH had not regressed 8 weeks after RTx. Representative B-mode images showed ventricular wall thickening in SNx animals and SNx + Tx groups compared to sham controls (Figures 4F–I). However, despite only partial reversal of LVH, metric evaluation of parasternal long-axis echocardiographic images measured in M-mode showed volume overload in both systole (Figure 4J) and diastole (Figure 4K) in the SNx group compared to the Sham and Tx groups. This is even more significant in the linear evaluation comparing systole and diastole during cardiac activity (Figure 4L). Volume overload resulted in dysfunction in the SNx group, as evidenced by a trend toward a reduction in fractional shortening (Figure 4N) and ejection fraction (Figure 4M). Myocardial measurements, shown here as a surrogate for volume measurements, also showed ventricular dilation in the SNx group compared to the sham and Tx groups (Figure 4O).

**Figure 5:**
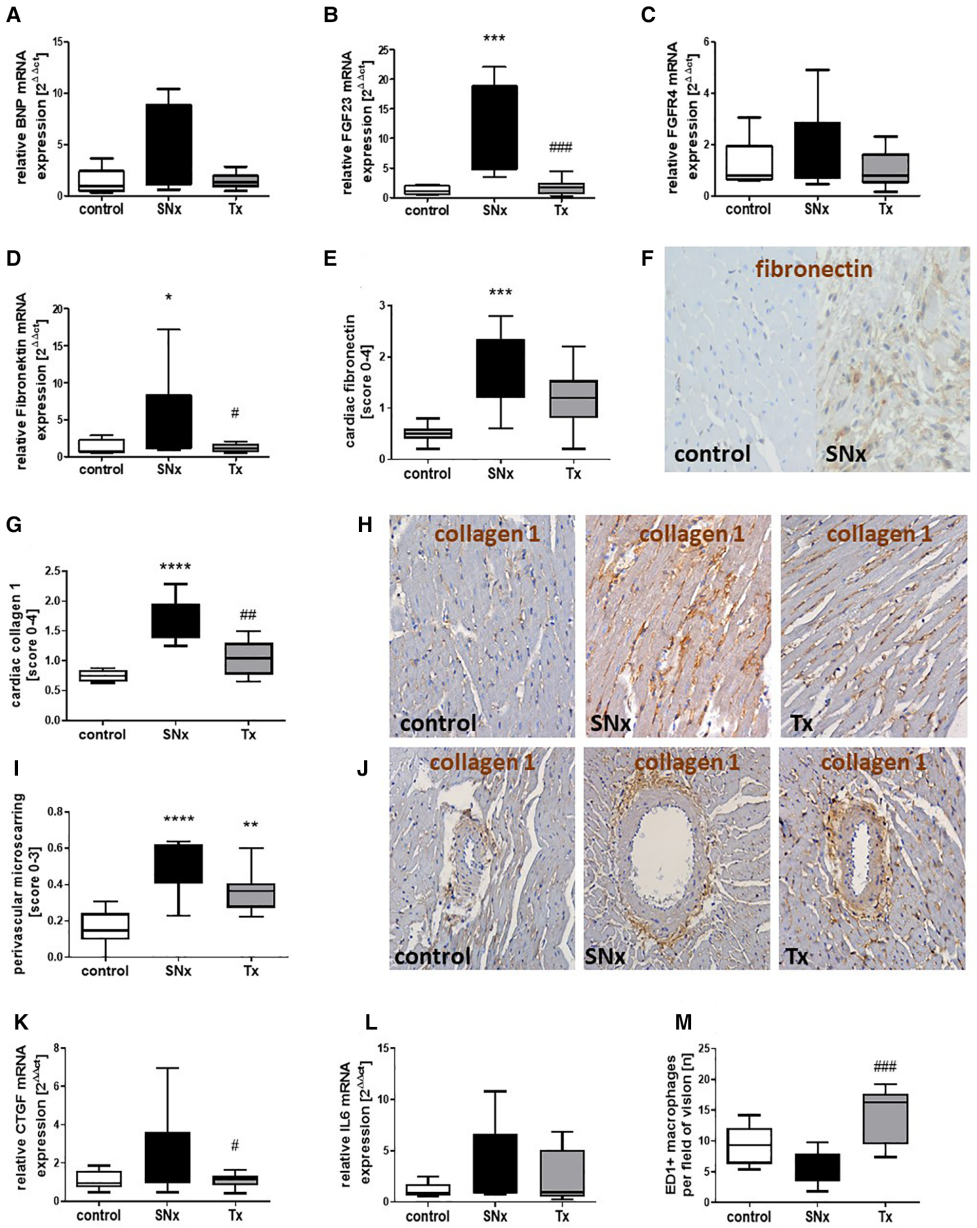
Changes in markers of cardiac hypertrophy, fibrosis and inflammation in CKD and after renal function restoration by transplantation. mRNA expression of BNP (**A**), FGF23 (**B**), FGFR4 (**C**), fibronectin (**D**), CTGF (**K**) and IL-6 (**L**) was analyzed in hearts from sham, SNx and SNx rat with renal transplant using Real-time PCR. Cardiac fibronectin (**E**) was analyzed by immunohistochemistry using a semi-quantitative score and examples of cardiac fibronectin staining in controls and SNx rats are shown (**F**, brown staining). The myofibroblast marker collagen 1 was analyzed semi-quantitatively in heart sections (**G**). Representative pictures of collagen staining in hearts of sham control, SNx and RTx rats are shown (**H**, brown staining). Perivascular microscarring was analyzed using a semi-quantitative score (**I**) and collagen 1 stained sections (**J**). Cardiac macrophages were analyzed by counting of ED1-positive cells detected using immunohistochemistry (**M**). *p < 0.05 vs. control, **p < 0.01 vs. control, ***p < 0.001 vs. control, ****p < 0.0001, ^#^*p* < 0.05 vs. SNx, ^##^*p* < 0.01 vs. SNx, ^###^*p* < 0.001 vs. SNx.

In addition to echocardiographic studies at the end of the study, we also analyzed cardiac gene expression of targets involved in either cardiac hypertrophy or fibrosis development using real-time PCR. Hypertrophy-inducing BNP was expressed at equally low levels in both sham controls and the transplanted SNx group (Figure 5A). In the heart of SNx animals, BNP expression was significantly increased in many animals, but showed a very high variance so that no significant differences were observed (Figure 5A). FGF23, a growth hormone also involved in mediating cardiac hypertrophy, was also expressed at low levels similar to BNP in the hearts of sham and kidney transplanted SNx rats (Figure 5B). In contrast, FGF23 mRNA expression was upregulated on average 10-fold in SNx animals at week 16 compared to the sham control group and the RTx group (Figure 5B). In contrast, the expression of FGFR4, which is thought to be responsible for FGF23-mediated hypertrophy, did not differ between groups (Figure 5C). Fibronectin mRNA expression, a marker of cardiac fibrosis, was also significantly increased in the SNx group compared with the control and RTx groups (Figure 5D). Immunohistochemical staining of fibronectin confirmed the significant upregulation in the SNx group compared with controls, but cardiac fibronectin was still tending to be elevated after RTx (Figures 5E,F). In addition, staining for the myofibroblast marker collagen 1 was significantly increased in SNx rats and also normalized by renal transplantation (Figures 5G,H). Perivascular microscarring was low in all heart sections but significantly increased in SNx. In the RTx group, mean perivascular scarring was lower compared to SNx group, but did not reach the significance level due to high variance (Figures 5I,J). The mRNA expression of the pro-fibrotic growth factor CTGF showed a similar pattern to that of fibronectin with an upregulation restricted to the SNx group (Figure 5K). Cardiac IL-6 showed the greatest variance in the SNx group, but was also expressed with a higher variance compared to the control after kidney transplantation (Figure 5L); the significance level was not reached for any comparison. Finally, macrophage infiltration in the hearts was examined. Interestingly, we tended to detect more macrophages in the hearts of control animals and significantly more macrophages in rats with transplantation compared to the SNx group (Figure 5M).

## 4. Discussion

In our study, we asked whether CKD-induced cardiovascular changes could be arrested or even reversed in their development by improving renal function after transplantation. Here, we showed that RTx reversed left ventricular wall thickening and normalized cardiac function in the rat model. However, measurement of relative heart weight and echocardiographic evaluation of left ventricular mass showed that LVH was not completely reversed 8 weeks after kidney transplantation. We cannot explain well the discrepancy between the reduced ventricular wall thickness seen in histological sections after RTx and the persistent LVH in terms of heart weight and ventricular mass determined by echocardiography. However, it is possible that wall thickness is already decreasing while the heart is still enlarged. In humans, there are several studies using echocardiographic or magnetic resonance techniques to investigate the effects of renal transplantation on LVH and cardiac function. In most of these studies, renal transplantation reduced LVH and left ventricular mass (LVM) compared to pre-transplantation levels, independent of blood pressure (11, 12, 14, 21, 22). Compared to these human studies LVH reversal was less pronounced in the rat CKD model. However, improvement of LVH after renal transplantation was not seen in all human studies. For example, in the study of Mitsnefes et al., renal transplantation had no effect on LVH, but LVH was dependent on blood pressure (16) and another study also reported no changes in LVH after kidney transplantation (15). In addition to the echocardiographic studies, we investigated FGF23 levels in our animal model, which are thought to be important for the development of LVH (9). FGF23 is mainly produced by osteocytes in healthy individuals, but in patients with CKD FGF23 is also expressed in the kidney and heart, resulting in significantly elevated serum levels. Therefore, we were able to detect significantly elevated FGF23 plasma levels in our animal model as early as four weeks after induction of CKD. Transplantation was able to restore cardiac expression of FGF23 to control levels. Although another study showed that inhibition of FGF23-induced cardiovascular changes by specifically blocking the FGF23/FGFR4 interaction reversed LVH in the rat 5/6 nephrectomy model (23), in our study LVH was not reversed after normalization of FGF23 due to transplantation. However, in the FGFR4 blockade study, treatment was also given as early as 4 weeks after SNx (24). It is possible that cardiac hypertrophy was more advanced in our experiment and therefore could not be reversed as quickly. In the human studies describing regression of LVH after transplantation, LVH was also assessed significantly later than in our study, i.e., between 6 and 19 months (11, 14, 21, 22). Therefore, later regression of LVH in our model cannot be excluded, especially since cardiac function and hypertrophy markers like BNP have already normalized. Normalization of cardiac function and molecular markers of hypertrophy suggest that these changes occur first in the reversal of LVH, whereas regression of morphologic changes appears to take longer.

In addition to hypertrophy, fibrosis also develops in the hearts of patients with CKD (25) which we also saw in our SNx model. Fibronectin, as a fibrosis marker, was significantly upregulated at both the mRNA and protein levels in the hearts of SNx rats. Interestingly, although fibronectin mRNA was reduced to control levels by kidney transplantation, it still tended to be elevated when the protein was detected by immunohistochemistry. This suggests that matrix molecules may have a relatively long half-life and may be detectable at elevated levels for even longer, depending on the extent of fibrosis. However, the myofibroblast marker collagen 1 was almost completely normalized in hearts from rat receiving a renal transplant. Cardiac fibrosis may be directly induced by high blood phosphate levels occurring in CKD and mediated by CTGF. This pro-fibrotic growth factor was also upregulated in hearts from SNx rats and restored after renal transplantation. *In vitro* experiments demonstrated that cardiac fibroblasts showed increased production of CTGF after stimulation with high Pi media (26). On the other hand, cardiac fibrosis may also be mediated or at least supported by the above mentioned FGF23. In models of myocardial infarction and cardiac ischemia/reperfusion, FGF23 has been shown to promote cardiac fibrosis via a beta-catenin-mediated process (23).

Inflammatory responses are also a possible trigger for cardiovascular changes in CKD. An association between the inflammatory response in CKD and cardiac geometry was also hypothesized and confirmed in the Chronic Renal Insufficiency Cohort (CRIC) study. In addition to hs-CRP, plasma levels of TNF-alpha, IL-1RA and IL-6 were measured and compared with echocardiographic values. It was clearly demonstrated that plasma levels of hs-CRP and IL-6 were associated with LVH (27). In SNx mice, salt-induced inflammation and fibrosis could be blocked by inhibition of IL-6 (28), suggesting that this interleukin is also an important mediator in cardiorenal syndrome. In our study, we were able to show that in CKD IL-6 mRNA also tends to be upregulated in the heart, which seems to be reduced after kidney transplantation. Thus, LVH may also be induced by IL-6 produced locally in the heart. However, it is likely that IL-6 is also produced in the inflammatory response of the chronically altered kidney and reaches the heart via the circulation. LVH may be mediated by FGF23 rather than directly by the inflammatory stimuli. *In vitro* studies have shown that IL-6, TNFα, or LPS can upregulate FGF23 mRNA and protein in primary neonatal and adult mouse cardiac fibroblasts (10). ED1-positive cardiac macrophages tended to be decreased in the SNx group compared to the healthy control group, but were significantly increased in the kidney transplant group. This finding is very surprising, as cardiac macrophages are thought to play a major role in remodeling. Thus, macrophage depletion significantly reduced LVH in salt-sensitive salt-fed rats and in mice subjected to transverse aortic constriction and pressure overload (29). There is also evidence for a protective role resident macrophages in cardiac remodeling (30). Therefore, the role of cardiac macrophages in our CKD model remains unclear, but was influenced by renal function.

The relatively short observation period is a clear limitation of our study. In fact, we started with experiments of longer duration. We stopped these studies because with increasing duration of 5/6 nephrectomy, many non-transplanted animals died and only those with very moderate renal injury and thus moderate cardiac changes survived. Thus, the differences in LVH at the end were not more pronounced than in the experiments of this study with a total duration of 16 weeks. However, the results of these preliminary study confirm our results (Supplementary Figure S1). In this study, we focused on few potential factors involved in mediating cardiovascular changes in chronic kidney disease. This is another limitation of the study, because the interaction between the kidney and the heart is very complex (31). Among the possible mechanisms of how chronic kidney disease mediates cardiovascular changes is endothelial dysfunction (31), which can be mediated by various uremic toxins such as asymmetric dimethylarginine (ADMA) (32), oxidative stress (33), and inflammation (27). Whether these factors are also upregulated in our animal model of CKD and restored to normal after kidney transplantation needs to be clarified in further studies.

Taken together, our results show that renal transplantation can reverse CKD-induced changes in cardiac output, function and fibrosis by normalizing renal function. This appears to be mediated by restoring cardiovascular triggers such as FGF23, CTGF, and IL-6 to controlled levels. However, LVH was not completely reversed during the observation period of 8 weeks after kidney transplantation. Thus, kidney transplantation probably contributes significantly to the risk reduction for cardiovascular events. Further long-term studies are needed to determine whether restoration of renal function leads to complete regression of LVH in the long term, as suggested by some human studies, even when marked cardiovascular changes are already present.

## Data Availability

The original contributions presented in the study are included in the article/**Supplementary Material**, further inquiries can be directed to the corresponding author.
