# Supplementary material for: Experimental renal transplantation in rats improves cardiac dysfunction caused by chronic kidney disease while LVH persists
Source: Front Cardiovasc Med. 2023 Jun 29;10:1200323. doi: 10.3389/fcvm.2023.1200323 (PMC10340545; doi:10.3389/fcvm.2023.1200323)
Supplement: Supplementary file 2 [file Datasheet1.docx]

Supplementary Material

Experimental renal transplantation in rats improves cardiac dysfunction caused by chronic kidney disease while LVH persists

Linda Hagmayer^1^, Christina Mayer^1^, Nadja Ebert^1^, Kerstin Amann^1^, Christoph Daniel^1*^

^1^Department of Nephropathology, Friedrich-Alexander University (FAU) Erlangen- Nuremberg, Germany

**
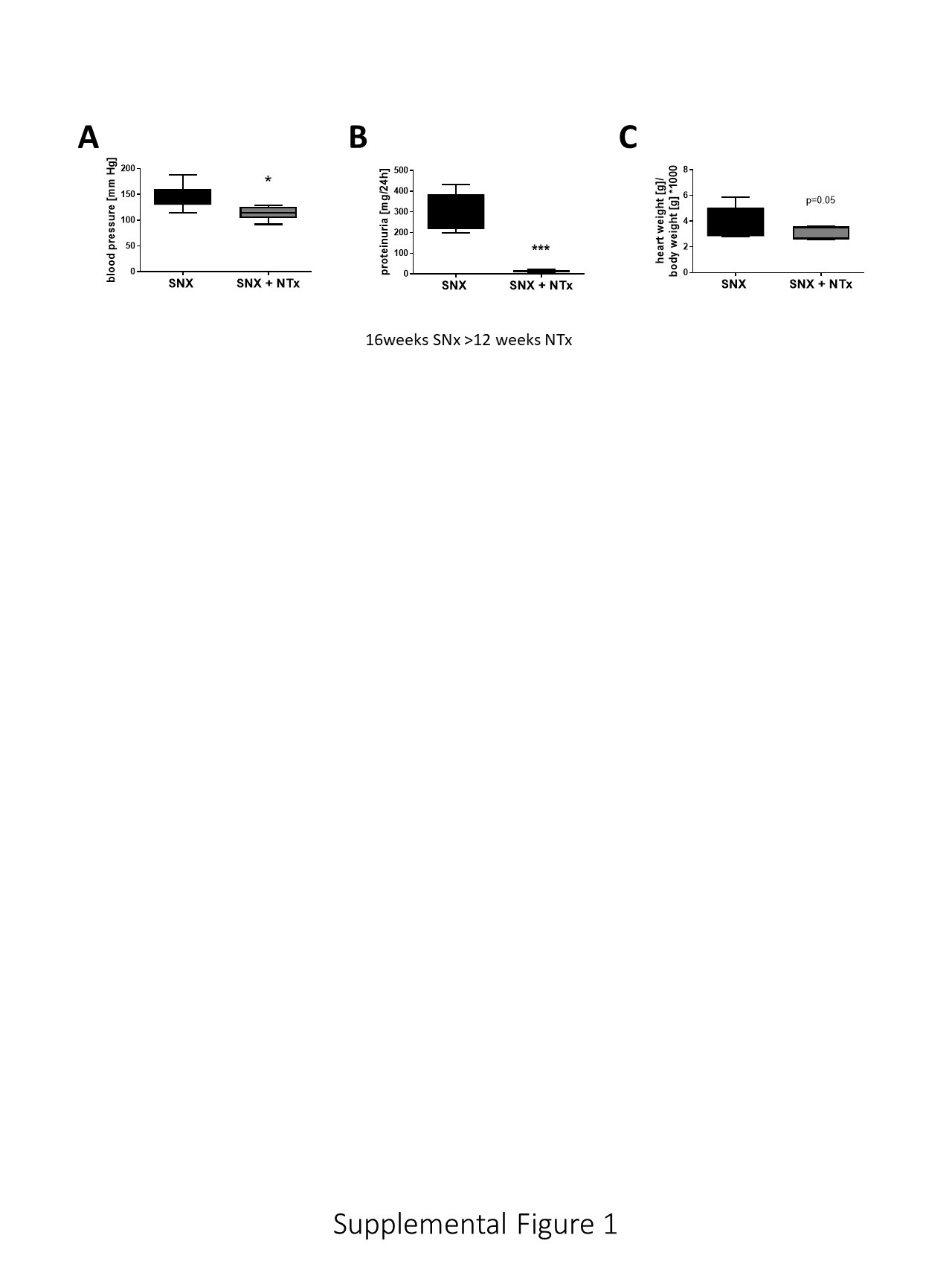
**

**Supplemental Figure 1: Changes in blood pressure, proteinuria and relative heart weight after SNx followed by kidney transplantation.** In a preliminary experiment, F344 rats underwent 5/6 nephrectomy (SNx, n=11) for 16 weeks and renal function was subsequently restored in a proportion of the animals by renal transplantation (RTx, n=6). Twelve weeks after RTx, the experiment was terminated and intra-arterial blood pressure (A), proteinuria (B) and relative heart weight (C) were determined at the end of the experiment.
